# The complete mitochondrial genome of a grasshopper endemic to the Qinghai-Tibet plateau, *Uvaroviola multispinosa* (Acrididae: Oedipodinae)

**DOI:** 10.1080/23802359.2019.1660247

**Published:** 2019-09-02

**Authors:** Bing Yu, De-Long Guan, Sheng-Quan Xu

**Affiliations:** a College of Biological and Environmental Engineering, Xi'an University, Shaanxi Province, Xi’an, P. R. China;; b College of Life Sciences, Shaanxi Normal University, Shaanxi Province, Xi’an, P. R. China

**Keywords:** *Uvaroviola multispinosa*, grasshopper, mitochondrial DNA, molecular marker, data mining

## Abstract

The grasshopper *Uvaroviola multispinosa* (Acrididae: Oedipodinae), is an agricultural pest to pasture and limitedly distributed in Qinghai-Tibet plateau of China. The complete mitochondrial genome of *U. multispinosa* is 15,620 bp long, which comprises of 13 protein-coding genes (PCGs), two ribosomal RNA genes, 22 transfer RNA (tRNA) genes and a putative non-coding control region (GenBank accession ID: MK829651). These genes are unequally distributed on different DNA chains, which 23 are located on the majority Chain. The nucleotide composition shows evidently bias (A, C, G, and T was 43.8, 14.8, 10.1, and 31.3%, respectively) with an overall AT content of 75.2%. All PCGs are initiated by ATN codons, among them ATG is the most preferred. Eleven PCGs use a common stop codon of TAA or TAG, whereas the remaining two were terminated with single T as incomplete stop codon. The phylogenetic relationships based on Bayes method showed that *U. multispinosa* is closely related to *Compsorhipis davidiana*, which is in accordance with its traditional morphological classification.

The grasshopper *Uvaroviola multispinosa* (Acrididae: Oedipodinae) is endemic to Qinghai-Tibet plateau of China (Gong and Zheng 2003). They feed on pasture, therefore was treated as agricultural pest. As a type of insect that has great adaptability to high altitude, this species has attracted much attention in study their unique features of population and evolution. However, as the most valuable data which could contribute to these studies, the molecular sequences are missing for this species. Thus, in this study we have reported the first complete mitochondrial genome of *U. multispinosa*.

The specimen of *U. multispinosa* used in this study were collected from Qinghai Province (E: 100°13′, W: 36°08′), China and preserved in the insect specimen museum of Shaanxi Normal University (Shaanxi province, China) under the voucher number of UVMU2018. The assembly for the mitochondrial genome was conducted from 3.39 Gb of Illumina Hiseq 2500 Pair-end sequencing data using the Mira/mitobim pipeline (Han et al. 2014; Machado et al. 2016). The published *Bryodema miramae miramae* mitochondrion complete genome sequence (Genbank ID: KP889242) was employed as a reference during the assembly.

The complete mitogenome of *U. multispinosa* is a closed-circular molecule of 15,620 bp in length (The sequence was submitted to GenBank under the accession of MK829651), and containing the typical set of 13 protein-coding genes (PCGs), two ribosomal RNA genes (rrnL and rrnS), 22 transfer RNA genes (tRNAs), and a putative non-coding control region (Boore 1999). The gene order and organization of *U. multispinosa* are consistent with those of putative ancestor of grasshopper, which the location of trnD and trnK was re-arranged and thus different as observed in other insects (Zhao 2010). The nucleotide composition of the mitogenome of *U. multispinosa* is evidently biased with A, C, G, and T content of 43.8, 14.8, 10.1, and 31.3%, respectively. The AT-skew and GC-skew of this genome were 1.39 and 0.68, respectively. This genome has great bias towards AT which the overall AT contents is 75.2%. The length of PCGs is 11,205 bp, which takes 71.7% of the total length. The length of 22 tRNAs varies at small range which among 64 bp (trnT and trnP) to 71 bp (trnV), the total length is 1,469 bp. Two rRNAs (rrnL and rrnS) are 1305 bp and 782 bp, respectively. They are not adjacent and trnV is inserted between them.

As for the coding sequences, their initial codons are all typical ATN (ATG for cytb, atp6, cox2, cox3, nad2, nad4, nad4l, nad6; ATT for nad3, nad5; ATA for nad1; ATC for atp8 and cox1). ATG is the most commonly used start codon. The typical termination codon (TAA or TAG) occurs in 11 PCGs, and only two PCGs (nad2 and nad5) are terminated with a single T as an incomplete stop codon. Based on the concatenated amino acid sequences of 13 PCGs, the Bayes method (Xu et al. 2003; Cox et al. 2004; Lumbsch et al. 2004) was used to construct the phylogenetic relationship of *U. multispinosa* with 14 other grasshoppers. The result supported that *U. multispinosa* is closely related to *Compsorhipis davidiana* (Figure 1), which is in accordance with the traditional morphological classification (Schlösser et al. 2010).

**Figure 1.**
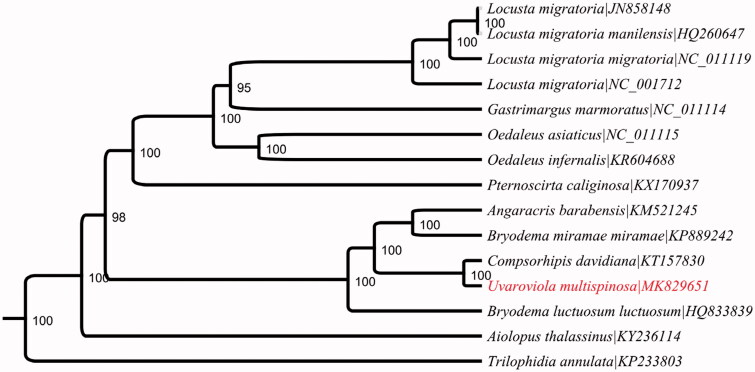
The phylogenetic tree conducted using our reported sequence and 14 published sequences from Oedipodinae sub-family. The UPGMA method was used.
